# PCSK9 Induces Tissue Factor Expression by Activation of TLR4/NFkB Signaling

**DOI:** 10.3390/ijms222312640

**Published:** 2021-11-23

**Authors:** Valentina Scalise, Chiara Sanguinetti, Tommaso Neri, Silvana Cianchetti, Michele Lai, Vittoria Carnicelli, Alessandro Celi, Roberto Pedrinelli

**Affiliations:** 1 Dipartimento di Patologia Chirurgica, Medica, Molecolare e dell’Area Critica, Università di Pisa, 56126 Pisa, Italy; chiara.sanguinetti@phd.unipi.it (C.S.); tommaso.neri79@for.unipi.it (T.N.); silvana.cianchetti@unipi.it (S.C.); vittoria.carnicelli@unipi.it (V.C.); alessandro.celi@unipi.it (A.C.); roberto.pedrinelli@unipi.it (R.P.); 2 Dipartimento di Ricerca Traslazionale e delle Nuove Tecnologie in Medicina e Chirurgia, Università di Pisa, 56126 Pisa, Italy; michele.lai@unipi.it

**Keywords:** PCSK9, tissue factor, TLR4, NFkB, inflammation, quantitative confocal microscopy

## Abstract

Proprotein convertase subtilisin kexin 9 (PCSK9) increases LDL cholesterol (C) concentration by accelerating the hepatic degradation of the LDL receptor (R) thus promoting atherogenesis. The molecule, however, also exerts proinflammatory effects independent of circulating LDL-C by enhancing local cytokine production and activation of NFkB, a process that might involve Toll-like receptor 4 (TLR4), a crucial component of the innate immunity system. Tissue factor (TF), a glycoprotein which plays an essential role in coagulation and inflammation, is rapidly induced by circulating monocytes stimulated by proinflammatory agents through NFkB-dependent mechanisms. The aims of our study were (1) to assess whether PCSK9 may induce monocytic TF expression and (2) to evaluate whether the TLR4/NFkB signaling pathway may contribute to that effect. Experiments were carried out in peripheral blood mononuclear cells (PBMCs), THP-1 cells, and HEK293 cells transfected with plasmids encoding the human TLR4 complex. PCSK9 increased procoagulant activity (PCA), mRNA and TF protein expression in both PBMCs and THP-1 cultures. Pre-treatment with inhibitors of TLR4/NFkB signaling such as LPS-RS, CLI-095, and BAY 11-7082, downregulated PCSK9-induced TF expression. A similar effect was obtained by incubating cell cultures with anti-PCSK9 human monoclonal antibody. In TLR4-HEK293 cells, PCSK9 activated the TLR4/NFkB signaling pathway to an extent comparable to LPS, the specific agonist of TLR4s and quantitative confocal microscopy documented the colocalization of PCSK9 and TLR4s. In conclusion, PCSK9 induces TF expression through activation of TLR4/NFkB signaling.

## 1. Introduction

Proprotein convertase subtilisin kexin 9 (PCSK9) is a 74 kDa serine protease consisting of 692 amino acids. The enzyme is composed of a signaling peptide (aa 1–30), prodomain (aa 31–152), catalytic domain (aa 153–404), hinge region (aa 405–454), and the C-terminal domain rich in cysteine and histidine (aa 452–692) [1]. PCSK9 controls plasma cholesterol (C) levels by accelerating the hepatic degradation of the LDLR, thereby preventing clearance of LDL-C [1] and, through that mechanism, promotes atherogenesis [2]. In agreement with this concept, PCSK9 inhibition by human monoclonal antibodies decreased drastically LDL-C and prevented the clinical sequelae of atherosclerotic vascular disease [3]. PCSK9, however, also exerts proinflammatory effects independent of circulating LDL-C by enhancing local cytokine production [4] through activation of nuclear factor kappa B (NFkB), a ubiquitous transcription factor key in the expression of cytokine and immune response genes [5]. Recent data also indicate that the proinflammatory effects of PCSK9 are dependent upon toll-like receptor 4 (TLR4) signaling [6,7,8,9], a crucial component of the innate immunity system which recognizes pathogen-associated molecular patterns from bacteria, virus, fungi as well as a variety of host molecules [10].

TF, a cell-surface single chain glycoprotein related to the cytokine receptor class II family, is the cellular receptor and essential cofactor of the coagulation factors FVII and FVIIa and, as such, plays an essential role in coagulation and thrombosis [11]. TF is expressed constitutively by many tissues not in contact with blood and is also rapidly induced by macrophages and monocytes primed by inflammatory cytokines and microbic products, a mechanism controlled at the transcriptional level by NFkB [12]. TF expression in concert with other factors of the extrinsic coagulation pathway also contributes to the amplification of inflammatory responses [13] since coagulation and inflammation are closely interrelated phenomena [14].

On that background, we hypothesized that PCSK9 might induce TF expression as a function of its proinflammatory properties [4], and that the TLR4/NFkB signaling pathway might act as a transducer of that response [6,7,8,9]. For that reason, we carried out this study in peripheral blood mononuclear cells (PBMC)s, THP1 cells, HEK-Blue hTLR4, HEK-Blue Null2 cells and HEK293-hTLR4-GFP, stable cell lines transfected with plasmids generated by fusing the green fluorescent protein (GFP) gene to the C terminus of hTLR4 gene.

## 2. Results

### 2.1. Effect of hrPCSK9 on TF Expression in PBMCs

**Figure 1:**
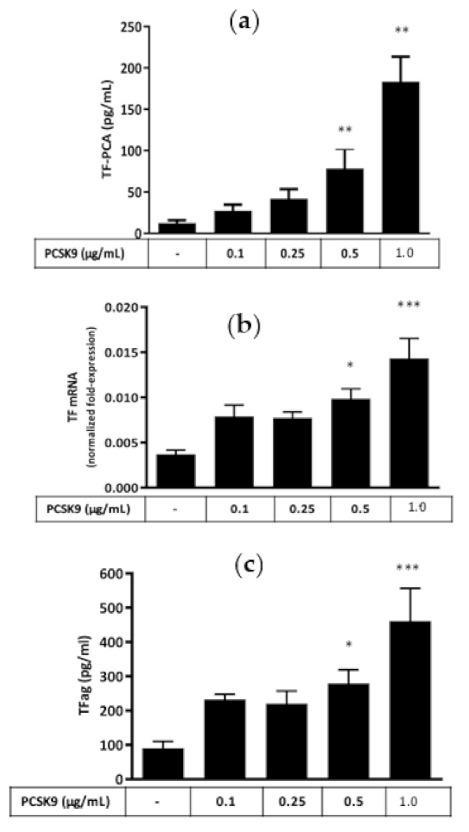
hrPCSK9 stimulates TF expression in human PBMCs. Concentration-dependent stimulation of TF PCA (*n* = 7, Panel (**a**)), mRNA (*n* = 6, Panel (**b**)) and ag (*n* = 7, Panel (**c**)) in response to hrPCSK9. * *p* < 0.05, ** *p* < 0.01, *** *p* < 0.001 vs. baseline. Mean ± SEM.

**Figure 2:**
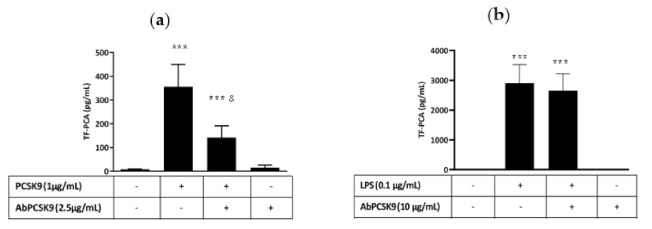
Anti-PCSK9 mAb downregulates TF activity in human PBMCs. Anti-PCSK9 mAb inhibits hrPCSK9-induced TF PCA (*n* = 8, Panel (**a**)) in PBMCs without affecting LPS-induced TF PCA (*n* = 7, Panel (**b**)). *** *p* < 0.001 vs. baseline, and & *p* < 0.001 vs. PCSK9. Mean ± SEM.

TF PCA (Figure 1a), TF mRNA (Figure 1b) and TFag (Figure 1c) increased in a concentration dependent manner in response to hrPCSK9 reaching a maximum at 1.0 µg/mL without evidence of a plateau within the range of concentrations tested in our experiments. Pretreatment with a human anti-PCSK9 monoclonal antibody (mAb) inhibited PCSK9-induced TF PCA (Figure 2a) while leaving LPS-induced TF PCA unchanged (Figure 2b). Pretreatment with the anti-PCSK9 mAb per se did not affect baseline TF PCA.

### 2.2. Pharmacological Evaluation of the Involvement of TLR4/NFkB Signaling Pathway in PBMCs

**Figure 3:**
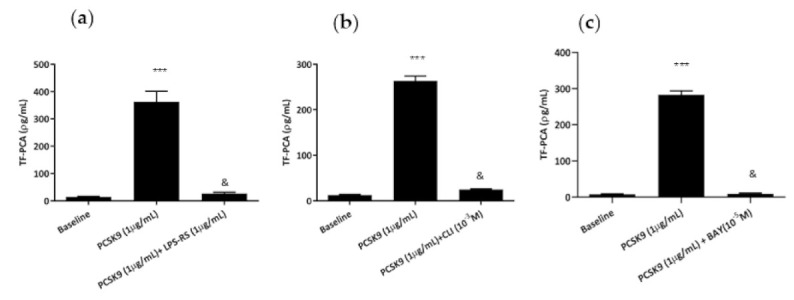
hrPCSK9-induced TF expression in human PBMCs is mediated through TLR4-NFkB signaling. Pharmacological antagonism of TLR4s by LPS-RS (Panel (**a**)) interference with downstream TLR4 signaling by CLI-095 (CLI, Panel (**b**)) and blockade of NFkB activation by BAY 11-7082 (BAY, Panel (**c**)) abrogates hrPCSK9-induced TF PCA stimulation in PBMCs. *** *p* < 0.001 vs. baseline, & *p* < 0.001 vs. hrPCSK9, *n* = 5 paired experiments per experimental groups. Mean ± SEM.

Blockade of TLR4s by LPS-RS [15], interference with TLR4 signaling through CLI-095 [16] and inhibition of NFkB by BAY 11-7082 [17] abrogated hrPCSK9-induced TF PCA stimulation (Figure 3a–c).

### 2.3. Effect of hrPCSK9 on TF Expression in THP-1 Cells

**Figure 4:**
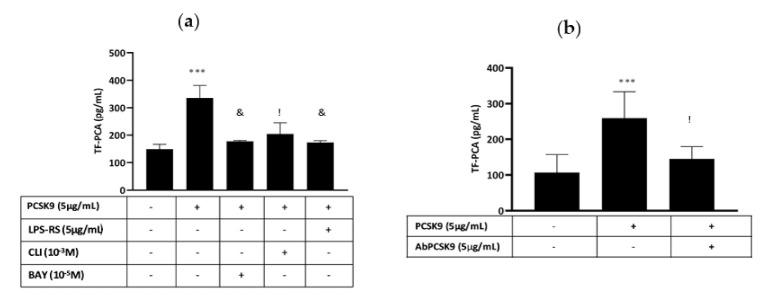
hrPCSK9 induces TF activation in THP-1 cells, a pure monocytic cell line. Panel (**a**): Stimulation of TF-PCA by hrPCSK9 and inhibition of that response by LPS-RS, CLI-095 (CLI) and BAY 11-7082 (BAY), *** *p* < 0.001 vs. baseline, ! *p* < 0.01 and & *p* < 0.001 vs. hrPCSK9, *n* = 5 paired experiments. Panel (**b**): Inhibition of PCSK9-induced TF PCA by anti-PCSK9 mAb. *** *p* < 0.001 vs baseline, ! *p* < 0.01 vs. hrPCSK9, *n* = 3 paired experiments. Mean ± SEM.

**Figure 5:**
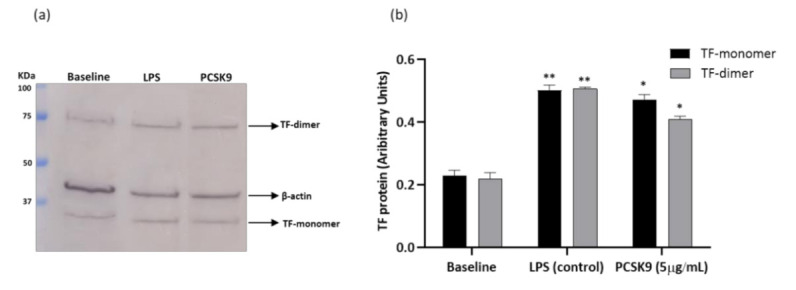
hrPCSK9 stimulates TF protein expression in THP-1 cells. Panel (**a**): Representative western blot image of the increased TF expression (monomer and dimer) in response to hrPCSK9 (5 µg/mL). Panel (**b**): Semi-quantitative densitometric analysis (ImageJ software) of TF monomer and dimer expression. Data reported as arbitrary units normalized to the corresponding β-Actin signals. LPS (1 µg/mL) was used as positive control. *n* = 3 independent series of western blot experiments, * *p* < 0.05, ** *p* < 0.01, vs baseline. Mean ± SEM.

THP-1 cells responded to hrPCSK9 stimulation by increasing TF PCA (Figure 4) and western blot analysis of TF protein confirmed the trend (Figure 5a,b). Pharmacological interference with the TLR4/NFkB signaling pathway (Figure 4a) as well as pretreatment with the human anti-PCSK9 mAb (Figure 4b) downregulated that response.

### 2.4. Effect of Anti-Human TF Antibodies on TF Expression

Incubation with an anti-TF antibody (20 µg/mL) confirmed that TF PCA stimulation in PBMCs as well as THP-1 cells was totally TF dependent (PBMCs TF PCA: from 283 ± 22 to 9 ± 4 pg/mL, *n* = 3, *p* < 0.01; THP-1 TF PCA: from 336 ± 128 to 40 ± 11 pg/mL, *n* = 3, *p* < 0.01).

### 2.5. Effect of NFkB/TLR4 Inhibitors on Cell Viability

PBMCs and THP-1 cultures with hrPCSK9 either *per se* or in presence of the various experimental interventions did not affect PBMCs or THP-1 cell viability (at least 90%, see method section).

### 2.6. hrPCSK9 Recognition by TLR4 in HEK293 Cells

**Figure 6:**
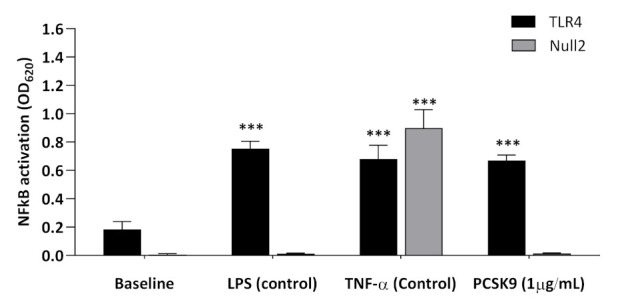
hrPCSK9 activates NFkB in hTLR4-positive HEK293 cell lines. Stimulation of NFkB expression by hrPCSK9 in hTLR4-positive (HEK-Blue-hTLR4) HEK293 cells stably expressing the cloned human hTLR4 receptor and its accessory proteins MD-2 and CD14 as opposed to TLR4-negative (HEK-Blue-Null2). LPS (0.1 µg/mL) and TNF-α (0.1 µg/mL) were used as a positive controls for responsiveness of both cells system. *** *p* < 0.001 vs baseline, *n* = 3 independent series of experiments. Mean ± SEM.

hrPCSK9 (1 µg/mL) activated NFkB in HEK-Blue-hTLR4 cell lines to an extent comparable to that obtained by LPS, the specific TLR4 ligand [18]. Both agonists induced no effect in HEK-Blue-Null2 cell lines (Figure 6) while NFkB stimulation was preserved in response to TNF-α (0.1 µg/mL), a cytokine acting independent of TLR4 signaling [19].

### 2.7. Immunolocalization of hrPCSK9 in HEK 293-hTLR4-GFP Stable Cell Lines

**Figure 7:**
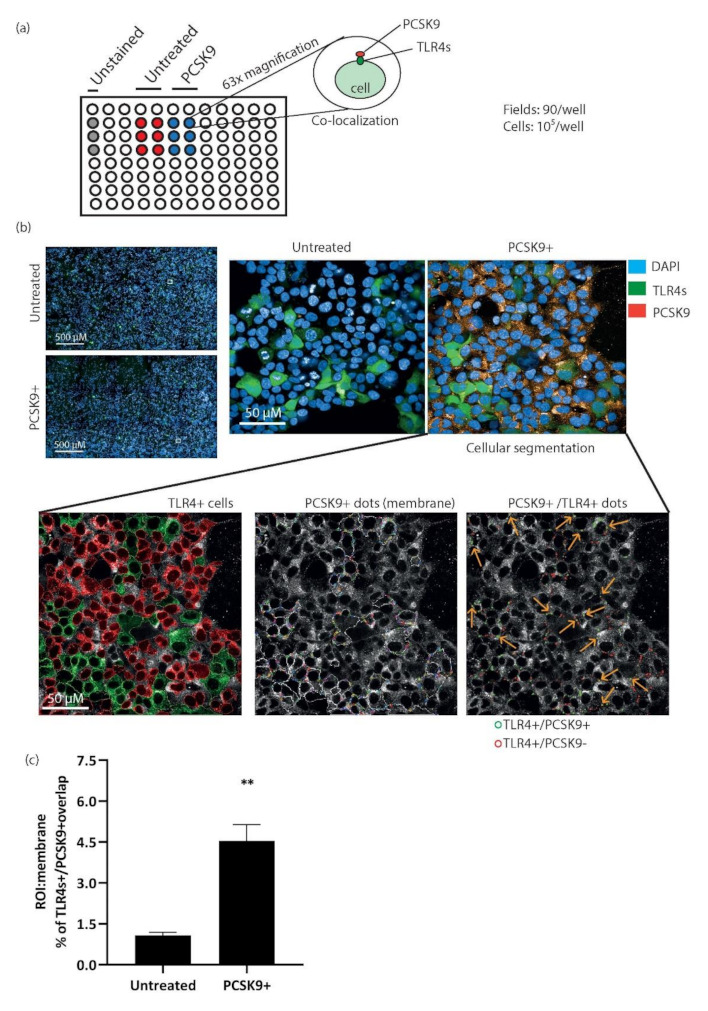
Co-localization of hrPCSK9 and TLR4 on HEK293 cell membrane. Panel (**a**): Diagram of the quantitative confocal microscopy experiments: 5 × 10^5^ HEK293-hTLR4-GFP cells were fixed and analyzed for TLR4s^+^/PCSK9^+^ overlapping puncta, using membrane as ROI. Panel (**b**): left panel: overview of 50 fields analyzed by high content screening. Center and right panel: representative images from left panel (white square) taken at 63× magnification. Bottom panels report segmentation analyses performed after acquisition. Briefly cells were segmented on TLR4^+^ and TLR4^−^. Then, TLR4^+^ cells were screened for TLR4^+^/PCSK9^+^ membrane spots and orange arrows indicate colocalization. Orange, green, and blue spots refer to hrPCSK9, TLR4s and nuclei respectively. Panel (**c**): Statistical analysis of TLR4s^+^/PCSK9^+^ colocalization. Mean ± SEM of two independent experiments with a least three replicates per experiments. ** *p* < 0.01 vs untreated.

To evaluate whether hrPCSK9 and TLR4s co-localize in HEK293 cultures, we took advantage of hTLR4-GFP-transfected cells, a cell line with negligible PCSK9 RNA content [20]. HEK293-hTLR4-GFP cells were treated with exogenous hrPCSK9 (1 ug/mL), then fixed and analyzed by high-content confocal screening. As schematically shown in Figure 7a, we counted the number of spot GFP^+^ (TLR4)/PCSK9 + (Alexa546) using the cell membrane as Region of Interest (ROI). The analysis, which comprised more than 80 fields and ~10^5^ cells/well revealed colocalization of hrPCSK9 and TLR4s **(**Figure 7b—orange arrows) in ~4% of membrane spots in the analyzed confocal plane (Figure 7c).

## 3. Discussion

The main and original findings of our study are twofold. First, we showed that hrPCSK9 stimulates TF PCA and protein expression in both human PBMCs and THP-1 monocytes and, second, we provided evidence for activation of the TLR4/NFkB signaling pathway as the biological transducer of the procoagulant action.

### 3.1. PCSK9 Upregulates Monocytic TF Expression

Stimulation of protein and mRNA expression and activation of PCA in PBMCs indicates that PCSK9 can induce de novo synthesis of functional TF in lysed PBMCs, a response wholly ascribed to TF/FVIIa interaction since an anti-TF antibody abolished that response. As reported graphically in Figure 1, hrPCSK9-stimulation of TF PCA in PBMCs exceeded by about three-fold the increase in protein and mRNA, an expected asymmetry explained by the increased availability of anionic phospholipids of our lysed PBMCs preparations [21]. It is important to note that PBMCs typically contain 25–35% monocytes in association with negligible proportions of neutrophils (<5%) and 65–75% lymphocytes, a cell line with no or, at its best, limited procoagulant potential [22] while procoagulant interferences from contaminating platelets [23] were excluded by absent procoagulant activity in clotting assays carried out in PBMCs-free preparations as verified in pilot experiments. Thus, stimulated TF expression in response to hrPCSK9 should be considered by and large a result of activated monocytes, a conclusion strengthened by TF PCA and protein stimulation in THP-1 cells, a human monocytic cell line without interfering platelets and lymphocytes. We took also great care in heating glassware at high temperatures and assessed on a routine basis LPS concentrations in reagents, solutions, and cell cultures through the chromogenic LAL assay [24]. Accordingly, contamination by LPS could be excluded in agreement with other authors who evaluated the proinflammatory effects of PCSK9 in human macrophages and THP-1 cells [25]. Finally, human anti-PCSK9 mAbs inhibited PCSK9-induced TF PCA in PBMCs without affecting the procoagulant effect of LPS, a specific TLR4 agonist [18], thus confirming the specific effect of PCSK9 on monocytic TF expression. It should be noted, in this regard, that downregulation of PCSK9-induced TF expression by anti-PCSK9 mAbs could only derive from disruption of the PCSK9/LDLR interaction since the Ab recognizes the LDLR EGF(A) binding site on the PCSK9 catalytic domain [26]. This consideration raises the issue of the involvement of LDLRs present on the surface of monocytic cells [27] as reported by others in different experimental conditions [25,28]. However, our study was not designed to address this specific point which needs further verification.

TF upregulation in response to hrPCSK9 implies a role of the protein in the coagulation process and provides a more precise understanding of an evolving concept based on still sparse evidence [29]. Thus, transgenic PCSK9^+/+^ murine models showed a systemic hypercoagulable environment reflected by elevated circulating thrombin/antithrombin complexes [30] while PCSK9^−/−^ mice developed less venous thrombosis after inferior vena cava ligation as compared with wild strains [31]. In man, hints in favor of the involvement of PCSK9 in coagulation come from the greater susceptibility to venous thrombosis among individuals with high anti-phospholipid antibodies titers in the presence of nucleotide polymorphisms of the PCSK9 gene [32]. A positive association between circulating PCSK9 and TF in patients with stable coronary artery disease [33] points in the same direction. Overall, the data are compatible with a role of circulating PCSK9 in the initiation of the coagulation process, particularly in conditions such as septic shock [34] in which high PCSK9 concentrations may prime circulating monocytes to express TF and initiate disseminated intravascular coagulation [35]. The procoagulant effect of PCSK9 may also play a pathogenetic role within the inflamed microenvironment of a disrupted atherosclerotic plaque by stimulating TF expression in resident macrophages favoring the formation of the occluding thrombus during acute coronary syndromes [4].

Stimulation of TF expression adds an additional facet to the proinflammatory profile of PCSK9 since TF expression is an essential component of the inflammatory response to invading agents [13,14]. As such, our data integrate previous reports showing PCSK9-induced stimulation of several cytokines such as TNF-alpha, interleukin-1β, -6, -12 from human and THP-1 macrophages [25]. Moreover, the formation of a TF/FVIIa complex is the initial step of a chain of enzymatic reactions leading to thrombin, a molecule endowed with well characterized bioregulatory and proinflammatory properties [13]

### 3.2. PCSK9 Activates TLR4/NFkB Signaling

TLR4s are transmembrane proteins defined by an extracellular domain for ligand recognition and a cytoplasmic domain which interacts with TLR signaling adaptor proteins allowing the downstream propagation of the biological signal resulting in NFkB activation [10], the critical factor for TF gene transcription [12]. Several exogenous and endogenous ligands bind the TLR4 and activate TLR4/NFkB signaling [10] and our experiments do suggest that PCSK9 stimulates TF expression through that same transduction pathway. In fact, LPS-RS, a competitive TLR4 receptor antagonist [15], CLI-095, a molecule that disrupts the interaction of endogenous adaptors to the TIR complex [16] and BAY 11-7082, a blocker of the NFkB activation [17], abrogated PCSK9-induced TF PCA in both PBMCs and THP-1 monocytes. As a matter of fact, the reduced sensitivity to PCSK9 stimulation of these latter is a feature already reported by others explained by an incomplete expression of the TLR4 signaling complex in these cells [36].

A next important result of our experiments was to show that hrPCSK9 activates NFkB by engaging the functional TLR4/MD-2/CD14 complex expressed by engineered HEK293 cells to an extent comparable to that obtained by LPS, the selective agonist of the TLR4 complex [18], likely through a mechanism independent of LDLRs since HEK293 cell lines do not express that receptor population [37]. As a counterproof, both hrPCSK9 and LPS did not activate NFkB in TLR4-negative HEK293 cell lines despite a maintained responsiveness of the system to TNF-alpha, a NFkB agonist acting through an independent signaling pathway [19]. Co-localization of PCSK9 and TLR4s on HEK293 cell membranes is a further support to our conclusions.

Several and concordant previous results agree with ours, including the antagonism exerted by CLI-095 on TF expression in response to oxidized LDL in PBMCs harvested from primates [38] as well as other studies conducted in highly heterogeneous experimental systems [6,7,8,9] per se suggestive of a more general underlying relationship. Quite important to note, PCSK9 shares a structural homology of its C-terminal cysteine-rich domain with resistin [39], a proinflammatory adipokine which binds TLR4s and activates TLR4 signaling through its C-terminal region [40], and induces expression of TF by NFkB mediated mechanisms [41]. That intriguing but coherent sequence of associations is unlikely to be merely coincidental. Moreover, the C-terminal cysteine-rich domain of resistin binds adenylyl cyclase-associated protein 1 [42], the same surface receptor that interacts with PCSK9 to escort the LDLR-PCSK9 complex towards lysosomal degradation [43]. Thus, the link between C-terminal cysteine-rich domain and two apparently unrelated molecules such as resistin and PCSK9 may perhaps offer a handle to understand how PCSK9 impacts on TLR4/NFkB signaling. However, further studies are needed to characterize more precisely the mechanisms involved in the recognition of PCSK9 by TLR4s in human monocytes.

In conclusion, hrPCSK9 stimulation elicits TF PCA and protein expression in both human PBMCs and THP-1 monocytes and activation of the TLR4/NFkB signaling pathway acts as the biological transducer of its procoagulant effect.

## 4. Materials and Methods

### 4.1. Reagent and Materials

RPMI-1640, DMEM, blasticidin, penicillin, streptomycin, trypan blue, Ficoll-Hypaque, sodium citrate, LPS from Escherichia coli O55:B5 applied without repurification, hrPCSK9, BAY 11-7082 (BAY), TF, TNF-alpha, β-actin, GAPDH and beta-2 microglobulin, oligodT primers were purchased from Sigma-Aldrich-Milan-Italy. Human anti-TF antibody (epitope specific for aa 1–25), TF antigen (ag) ELISA kit and relipidated full length recombinant human TF were obtained from BioMedica Diagnostics-Windsor-NS Canada. RNeasy mini kit and Quantitect Reverse Transcription Kit from Qiagen-Venlo-The Netherlands, Nanodrop from Thermo Fisher Scientific, Waltham, MA, USA, iQ5 Real Time PCR System, SsoAdvanced universal SYBR Green supermix, Mini-PROTEAN TGX Gel, Precision Plus Protein All Blue, Trans-Blot Turbo transfer system, Bio-Rad Opti-4CN substrate Kit, Goat Anti-Rabbit IgG H&L (HRP) and Goat Anti-Mouse IgG H&L (HRP) from Bio-Rad Laboratories, Hercules, CA, USA. Ultrapure LPS-RS, CLI-095 (CLI), HEK293 human (h)TLR4-positive (HEK-Blue hTLR4) and negative (HEK-Blue Null2) cell lines, primer sequences for qRT-PCR were purchased from InvivoGen-Toulouse-France. HEK293-hTLR4-GFP stable cell lines were purchased from Aurogene-Rome-Italy. Image-iT Fixation/Permeabilization Kit and goat anti-human IgG (H + L) secondary antibody Alexa Fluor Plus 568 were purchased from Invitrogen-Thermo Fisher Scientific, Waltham, MA, USA. Human recombinant ultrapure anti-PCSK9 mAb were kindly provided by Amgen Inc, Thousand Oaks, CA, USA. The mAb binds to an epitope which includes the LDLR EGF(A) domain binding site on the PCSK9 catalytic [25]. LAL chromogenic endpoint assay was obtained from Hycult Biotech-Uden-The Netherlands. All other chemicals were provided by the hospital pharmacy and were of the best grade available.

### 4.2. PBMCs Preparations

Human PBMCs suspensions were obtained from unpooled buffy coats left over from blood bank draws taken from healthy donors with the approval of the local ethics committee, kept at room temperature and utilized within a maximum of 4 hrs from withdrawal. PBMCs were isolated by centrifugation of fresh buffy coats on Histopaque-1077 at 400 xg for 30 min at controlled temperature of 20 °C. Cells collected from the interphase were washed twice in sodium citrate 0.38% and resuspended in RPMI-1640 medium supplemented with 1% penicillin-streptomycin. Drugs were kept in stock solution and diluted in serum-free RPMI at the appropriate concentrations immediately before use. Cell vitality by 3-(4,5-dimethylthiazol-2-yl)-2,5-diphenyltetrazolium bromide (MTT, >85% of viable cells) and constancy of cell number was verified throughout the experimental phases. The final PBMCs preparations typically contain 25–35 % monocytes, negligible proportions of neutrophils (<5%) and 65–75% lymphocytes and residual platelets [44]. After isolation, cells were resuspended in polypropylene tubes (3 × 10^6^ cells/mL) and pre-treated with the various pharmacological probes used in the study (see below) for 30 min prior to stimulation with hrPCSK9 or LPS and then left to incubate at 37 °C for 18 hrs for TF procoagulant activity (PCA) and ag determination. The longer incubation time of PBMCs compared to THP1 cells (see below) was due to the delayed delivery of buffy coats from the blood bank not allowing to complete the experimental procedures in the same day. In preliminary experiments, PBMCs responses to hrPCSK9 tested after 4 and 18 hrs incubation did not differ. TF mRNA quantification was carried out after 2 hr incubation.

### 4.3. THP-1 Preparations

To confirm the monocytic origin of TF, additional experiments were carried out in THP-1 cells, a human monocytic cell line derived from an acute monocytic leukemia patient. THP-1 cells, purchased from the European Collection of Authenticated Cell Cultures (ECACC, 88081201), were cultured in RPMI 1640 medium supplemented with 10% heat-inactivated FBS, 5% β-mercaptoethanol, 2 mM L-glutamine, 1% penicillin-streptomycin and incubated in a 5% CO2 humidified atmosphere at 37 °C. Cell counts and viability estimation by MTT were performed regularly. Throughout the study procedures, THP-1 cells were maintained in a logarithmic growth phase at a concentration of 3 − 5 × 10^5^ cells/mL. THP-1 cells were stimulated by hrPCSK9 for 4 h at 37 °C and exposed to the various pharmacological probes 30 min prior to stimulation.

### 4.4. TF PCA

PBMCs (3 × 10^6^ cells/mL in a 50 mL volume) and THP-1 cells (1×10^6^ cells/mL) were assayed for the capacity to shorten the spontaneous clotting time in a one stage clotting assay. Briefly, equal volume of pooled normal human plasma was added to PBMCs or THP-1 suspensions (100 µL) and then 25 mM CaCl_2_ was used to start thrombin generation at 37 °C. Experiments were run in triplicate and averaged. For each experimental session, calibration curves were generated using recombinant human relipidated TF (pg/mL) as a standard. Clotting times (means ± SD, *n* = 20 for step) by increasing TF concentrations were as follows: 0.001 pg/mL: 688 ± 84 sec; 0.01 pg/mL: 386 ± 56 sec; 0.1 pg/mL: 192 ± 29 sec; 1 pg/mL: 84 ± 6 sec; 10 pg/mL: 40 ± 3 sec; 100 pg/mL: 19 ± 2 sec. The interassay variation coefficient at each concentration step ranged from 7 to 15% (mean: 11%, 95% confidence interval: 7.5–14.7%). “Baseline” values refer to quiescent, non-activated, untreated cells with clotting times above 365 sec and 162 sec for PBMCs and THP-1 respectively.

### 4.5. TF ag Quantification

To provide a quantitative determination of total TF protein content of the lysed PBMCs, cells were disrupted by three repeated freeze–thaw cycles, debris pelleted by centrifugation at 100× *g* for 1 h at 4 °C and supernatants used for ELISA according to manufacturer’s instructions. Within and between assay variability was 3.5 and 5.5%, respectively.

### 4.6. TF mRNA Expression Analysis

Total RNA for gene expression analysis was extracted from PBMCs pellets (3 × 10^6^/mL) using the RNeasy mini kit. RNA concentration and purity were determined by optical density measurement via Nanodrop. A mixture of 0.5 ng total RNA per sample was retro-transcribed with random primer oligodT into cDNA using the Quantitect Reverse Transcription Kit. The retro-transcription cycle was performed at 25 °C for 5 min, 42 °C for 30 min and 95 °C for 3 min. RealTime-PCR was carried out in a iQ5 Real Time PCR System and iTaqUniversalSybr Green Supermix was employed on the basis of the manufacturer’s conditions: 95 °C, 30 s; 40 cycles 95 °C, 5 sec, 60 °C, 15 sec; a final melting protocol with ramping from 65 to 95 °C with 0.5 °C increments of 5 sec was performed. Primers were designed with Beacon Designer Software v.8.0 (Premier Biosoft International, Palo Alto, CA, USA) with a junction primer strategy. All samples were analyzed in duplicate and averaged. The relative expression of the target gene was normalized to the level of GAPDH and beta-2 microglobulin (B2B) in the same cDNA. Primer sequences used for qRT-PCR analysis were TF, sense 5′-TTGGCAAGGACTTAATTTATACAC-3′, antisense 5′-CTGTTCGGGAGGGAATCAC-3′; GAPDH, sense 5′-CCCTTCATTGACCTC AACTACATG-3′ and antisense 5′-TGGGATTTCCATTGATGACAAGC-3′; B2B sense 5′-GAGTATGCCTGCCGTGTG-3′ and antisense 5′-AATCCAAATGCGGCATCT-3.

### 4.7. Western Blot Analysis

For western blot determinations of TF, THP-1 cells were cultured and treated as describe above. After 4 h of incubation, cells were pelleted and resuspended in 25 µL of phosphate-buffered saline (PBS) and lysated on ice for 30 min. Protein concentration was determined using the Bradford assay [45]. Cell lysates were denaturated and fractionated by sodium dodecyl sulphate polyacrilamide gel electrophoresis (SDS-PAGE). For each sample, 50 μg of proteins were loaded on the 4–20% gradient Mini-PROTEAN TGX Gel and precision plus protein all blue was used as a standard. Separated proteins were transferred onto polyvinylidene difluoride (PVDF) membrane using a transfer apparatus (Trans-Blot Turbo) according to the manufacturer’s protocol. The membranes were blocked with 3% bovine serum albumin, incubated with the primary antibodies and the proteins detection were performed using the Bio-Rad Opti-4CN substrate Kit. After being washed three times, membranes were incubated with secondary antibodies goat anti-mouse IgG H&L (HRP) and goat anti-rabbit IgG H&L (HRP) (1:1000). Densitometry was performed using the open-source software ImageJ [46] The abundance of the TF protein was normalized to the total amount of the housekeeping protein (β-actin) in each lane, and relative expressions were calculated in comparison to the control (Baseline condition).

### 4.8. Cell Culture and TLR4 Signalling Assay

hTLR4-positive (HEK293-Blue-hTLR4) and TLR4-negative (HEK293-Blue-Null2) HEK293 cell lines were used to evaluate the engagement of TLR4s by hrPCSK9. The HEK293-Blue-hTLR4 cells are stably expressing the cloned human hTLR4 receptor and its accessory proteins MD-2 and CD14, while HEK293-Blue-Null2 cells lacking TLR4 were used as negative control. In these engineered cell line, the interaction of any ligand with the TLR4 complex induces NFkB activation to an extent proportional to the amount of secretion of functional SEAP protein into the cell supernatant. HEK293 cell culture was maintained according to manufacturer’s instructions. Shortly, the cells were harvested for stimulation as non-confluent (50–60%) and were plated at a cell density of 25 × 10^3^ cells/well in HEK-Blue detection medium in a flat-bottom 96-well plate and then incubated with or without stimuli for 16 h at 37 °C in a 5% CO2 humidified atmosphere. All experiments were done in triplicate. SEAP reporter activity was measured in the culture supernatant according to the manufacturer’s instructions, by reading the OD at 620 nm using a spectrophotometer plate reader.

### 4.9. Quantitative Confocal Microscopy

Co-localization of hrPCSK9 and TLR4s was further investigated in HEK293-hTLR4-GFP stable cell lines obtained by stable co-transfection of HEK293 cells with the pUNO1-hTLR4-GFP (Invivogen), a plasmid expressing the hTLR4 gene fused to a GFP gene. The cells were maintained in DMEM supplemented with 10% FBS, 1% penicillin/streptomycin, 2 mM L-Glutamine and containing blasticidin (7µg/mL) as a selection marker in a humidified incubator with 5% CO2 at 37 °C. The cells were allowed to adhere on 96-well Cell-carrier Ultra at density of 5 × 10^3^ /well. After 24 h the culture media was removed from each well and the cells were incubated with exogenous administration of hrPCSK9 for 15 min. Subsequently, the cells were washed three time with PBS with Ca^2+^ and Mg^2+^, taking great care to preserve the integrity of the monolayers and analyzed using an Image-iT Fixation/Permeabilization Kit. Briefly, cells were fixed for 15 min with 4% paraformaldehyde in PBS (pH7.3) at room temperature, then permeabilized with Triton X-100 0.5% for 15 min and blocked for 1 h with 3% BSA in DPBS (pH7.4). Cells were incubated with anti-PCSK9-mAb (1:50) for 1 h and 30 min at room temperature and then with goat anti-human IgG (H + L) secondary antibody Alexa Fluor Plus 568 (1:1000) for 1 h at room temperature for detection of hrPCSK9 localization. Nuclei were stained using DAPI and images acquired with Operetta CLS confocal fluorescent microscope (PerkinElmer, Hamburg, Germany). The analysis was performed using Harmony 4.6 software (PerkinElmer). PCSK9 spots were visualized in over 45 fields/well, taking images with 63× water objective and analyzing an average of 10^5^ transfected cells, as previously described [47]. Data were analyzed using the following building blocks: 1—Find Nuclei, 2—Find Cytoplasm of transfected cells (GFP^+^ Cells) 3—Find PCSK9^+^ spots, ROI: membrane 4—find GFP^+^ spots, ROI: membrane, 5—Calculate position properties, 6—Find % spots GFP^+^ overlapping with spots PCSK9^+^.

### 4.10. LPS Contamination

Since LPS contamination during the experimental procedures is frequent and leads to erroneous result interpretation [48], glassware was exposed to high temperature (200 °C for 4 h) to inactivate endotoxin by dry-heat treatment. In addition, plastic ware, reagents, and solutions used for in vitro cell cultures were preliminarily tested on a routine basis with a sensitive chromogenic LAL assay [24]. Only material with endotoxin concentrations <60 pg/mL, a concentration inactive on baseline TF expression, was used in the various experimental steps.

### 4.11. Statistical Analyses

The statistical significance of between- and among-groups differences was tested by Wilcoxon or Mann-Whitney rank sum test. Data were analyzed with GraphPad Prism 5 and expressed as mean ± SEM unless otherwise reported. *p* < 0.05 was considered as statistically significant.

## 5. Experimental Design

### 5.1. Effect of hrPCSK9 on TF Expression in PBMCs

To test the hypothesis that hrPCSK9 stimulates TF expression, TF PCA, ag and mRNA were assessed at baseline and in PBMCs exposed at increasing concentrations of hrPCSK9 (0.1, 0.25, 0.5, and 1.0 µg/mL) covering the spectrum of circulating PCSK9 ranging from normal to the highest levels reported in septic shock complicated by multiorgan failure [34]. The specificity of hrPCSK9-induced TF PCA stimulation in PBMCs was assessed by comparing the effect of anti-PCSK9 mAb in hrPCSK9 (1.0 µg/mL) or LPS (0.1 µg/mL)-stimulated cells.

### 5.2. Pharmacological Evaluation of the Involvement of TLR4/NFkB Signaling Pathway in PBMCs

TF PCA was assessed at baseline and in PBMCs primed by hrPCSK9 (1.0 µg/mL respectively) either in absence or presence of LPS-RS (1 µg/mL), a TLR4 receptor antagonist [15], CLI-095 (10^−3^ M), which affects recruitment of downstream TLR4 signal transduction [16], and BAY 11-7082 (10^−5^ M), a compound which inhibits NFkB by preventing the phosphorylation and subsequent degradation of IkB [17].

### 5.3. Effect of hrPCSK9 on TF Expression in THP-1 Cells

TF protein expression and PCA were analyzed at baseline conditions and after stimulation by hrPCSK9 (5 µg/mL) in THP-1. Moreover, PCA was measured in THP-1 primed by hrPCSK9 either in absence or after pretreatment with LPS-RS (5 µg/mL), CLI-095 (10^−3^ M), BAY 11-7082 (10^−5^ M) and anti-PCSK9 mAb (5 µg/mL). The lower sensitivity of THP-1 cells as compared to PBMCs agrees with previous studies showing a similar behavior of that cell line in response to LPS [36], the specific TLR4 ligand [18].

### 5.4. Effect of Anti-Human TF Antibodies on TF Expression

In separate series of experiments, both PBMCs and THP-1 cells were incubated with anti-human TF antibodies and a matched isotype (both at 20 µg/mL) as control to confirm the TF-dependency of PCA stimulation by hrPCSK9.

### 5.5. PCSK9 Recognition by TLR4 in HEK-Blue Cells

HEK-Blue cells were incubated either without or with hrPCSK9 (1 µg/mL) and LPS (0.1 µg/mL), the specific TLR4 ligand [18] and SEAP activity was measured as above described. To verify the maintained responsiveness of NFkB in TLR4-negative HEK293 (HEK-Blue-Null2), cell lines were exposed to TNF-alpha, (0.1 µg/mL), a cytokine which stimulates its own receptors independent of TLR4s [19].

### 5.6. Immunolocalization of PCSK9 in HEK293-hTLR4-GFP Stable Cell Lines

To detect physical proximity of exogenous hrPCSK9 (1 µg/mL) and hTLR4, we used HEK293-hTLR4-GFP stable cell lines and their colocalization was observed by Operetta CLS high-content imaging system by following the previously described procedures. Omission of the primary anti-PCSK9 antibody was used as a negative control.

## Data Availability

The datasets are available from the corresponding author on reasonable request.
